# Effects of the mental health promotion seminar ‘Coping with stress’ in the undergraduate medical curriculum of the Medical University of Vienna

**DOI:** 10.1186/s12909-023-05019-0

**Published:** 2024-01-08

**Authors:** Benedikt Till, Angelika Hofhansl, Thomas Niederkrotenthaler

**Affiliations:** 1 Unit Suicide Research & Mental Health Promotion, Department of Social and Preventive Medicine, Center for Public Health, Medical University of Vienna, Kinderspitalgasse 15, Vienna, A-1090 Austria; 2 Medical Didactics, Teaching Center, Medical University of Vienna, Vienna, Austria

**Keywords:** Stress management, Mental health, Distress, Burnout, Medical students

## Abstract

**Background:**

High prevalence rates of distress and burnout in medical students are well-documented in mental health literature. Different types of interventions have been developed in the past in order to reduce stress in medical undergraduate students and promote better coping skills. There is, however, a paucity of studies that have tested the effectiveness of these interventions. This study aimed to examine the effect of different versions of the seminar ‘Coping with stress’, which was implemented in the first year of the undergraduate curriculum of the Medical University of Vienna in the summer semester of 2018, on students’ mental health.

**Methods:**

Invitations to participate in the study were sent via email to six cohorts of students from the Medical University of Vienna. Two cohorts participated in the onsite version of the seminar ‘Coping with stress’, whereas two cohorts participated in the online version of the seminar, and two cohorts received no intervention (control group). Data on burnout risk, life satisfaction, stress, and knowledge about available help resources were collected via online questionnaires from *n* = 137 students before and after the curriculum module that contained the seminar.

**Results:**

Medical students who participated in the onsite seminar reported a reduction of some aspects of burnout, a decrease in stress, and an increase in knowledge about available help resources. No such effect was seen in the control group. Participants of the online seminar experienced a similar increase in knowledge about available help resources, but no changes in other outcomes.

**Conclusions:**

The findings support the notion that the onsite seminar of ‘Coping with stress’ had a positive impact on medical students’ mental health and is a useful addition to the medical curriculum by promoting mental health literacy.

**Trial registration:**

This research has been registered in the German Clinical Trial Registry with the registration number DRKS00018981 and the registration date 14/11/2019.

## Introduction

In the last few decades, alarming levels of professional and personal distress have been reported among primary care physicians [1]. This distress has been characterized with the term ‘burnout’ in the literature [2]. Burnout, originally described as syndrome of emotional exhaustion and cynicism [3], is commonly defined by its main symptoms, i.e., emotional exhaustion, depersonalization (e.g., treating patients as objects), and low sense of accomplishment [1, 4]. Although a prevalence of 50% for symptoms of burnout among physicians has been cited in academic literature, some studies have even reported a prevalence of up to 80.5% as indicated by respective scores on common questionnaires for burnout assessment [2], such as the Maslach Burnout Inventory [3]. Symptoms of burnout in medical professionals are associated with poorer quality of patient care, higher risk of medical errors and malpractice, as well as more stress-related health problems and lower life satisfaction [1, 2, 4, 5]. Furthermore, several studies found an increased suicide risk for physicians [6], particularly female physicians [7], which is often attributed to the job-related high stress levels [8].

Medical personnel face high levels of stress not only in their job, but also already throughout their education and training. In fact, stress exposure and mental health issues have been found to be greater among medical students than other student populations or age-matched individuals in the general population [9]. Performance pressure overload has been identified as one of the main reasons for stress in medical students [10]. A recent meta-analysis of studies that assessed burnout with psychometric measures found that prevalence of symptoms of burnout was 37.23% among medical students [9], and some studies even found assessment scores indicating symptoms of burnout in more than half of all medical students, resulting in a significantly higher prevalence rate for burnout in medical students than in other comparable populations [11, 12]. In several studies, medical students with symptoms of burnout have been found to preform worse in their professional development, place patients at risk, and report sleep disturbances [13, 14]. Experiencing symptoms of burnout is also associated with increased likelihood of suicidal ideation in medical students [11].

In order to decrease mental distress and improve psychological well-being in medical students, many mental health experts have highlighted the need to adopt preventive and intervention measures for medical students during training, e.g., by implementing programs to teach better coping skills [15–17]. The structure and content of these interventions vary and are sometimes based on relaxation training, mindfulness-based stress reduction, mentoring programs, stress management, or self-hypnosis, but little is known about the effectiveness of these interventions [18].

De Vibe and colleagues examined the effects of a voluntary seven-week mindfulness-based stress reduction program for medical and psychology students offered at two universities in Norway [5]. The authors found that the program reduced mental distress and improved subjective well-being in participants compared to a control group [5]. There was, however, no positive effect on symptoms of burnout. Participation in similar mindfulness-based training programs has been also associated with increased empathy and reduced anxiety, depression, mood disturbances, and psychological distress in previous studies [19–22]. In a more recent study, however, Kuhlmann et al. did not find differences in mental health outcomes between participants of a mindfulness-based stress prevention training for medical students, autogenic training, and a control group [18]. In a meta-analysis, stress prevention trainings were associated with moderate effects on medical students’ mental health, with interventions with a duration of 4 to 8 weeks exhibiting the largest effect sizes [23].

It is, however, important to note that most of these training programs are primarily mindfulness-based, and all of them are voluntary (i.e., not part of the medical curriculum). Studies on the effectiveness of other types of interventions targeting medical students (i.e., not based on mindfulness, integrated in the medical curriculum) are scarce. Furthermore, many studies that aimed to explore the effects of stress-reducing trainings for medical students had severe methodological limitations (e.g., no control group, poor statistical strength) [5, 18].

In the summer semester of 2018, the seminar ‘Coping with stress’ was implemented in the first year of the undergraduate medical curriculum of the Medical University of Vienna. The seminar had the objective to reduce distress and risk of burnout as well as improve mental health literacy and coping strategies in medical students. For the first time, a seminar on this topic was implemented as an obligatory part of the curriculum (as opposed to voluntary or optional seminars explored in previous studies [5, 18–22]). As a result, we were able to reach all students of the respective cohorts, including those who did not express any interest in improving their mental health literacy. Although the seminar included some basic elements of mindfulness, the seminar used predominantly conventional teaching techniques such as whole-class lectures and teacher-led instruction (as opposed to typical mindfulness techniques such as breath focus, body scanning, loving-kindness, and open monitoring of thoughts, feelings and bodily sensations [24]).

The seminar was designed as an onsite lecture, but due to the onset of the COVID-19 pandemic, an online version of the seminar was developed in 2020 and temporarily implemented in the curriculum. The onsite seminar was designed as a single lecture with a duration of 90 min. In order to accommodate the entire annual cohort of students, the lecture was taught in multiple parallel sessions (i.e., 33 individual sessions per cohort). All sessions were identical and accommodated 20–22 students. Thus, over the course of four weeks, the entire cohort completed the seminar. All discussions and group activities that were part of the seminar were conducted within these individual sessions consisting of 20–22 participants. In the online seminar, the students got access to selected materials on mental health literacy, had to reflect on their own coping strategies for prevention of distress and burnout in an essay, and received feedback on their essay by the respective instructor (but there were no lecture or group activities).

This adaptation enabled us to not only evaluate the effectiveness of the seminar per se, but also compare its different versions. Thus, in the current study, we investigated the impact of different versions of the seminar ‘Coping with stress’ on students’ mental health and mental health literacy. We hypothesized that the seminar will (a) reduce burnout risk, (b) increase life satisfaction, (c) decrease stress, and (d) improve knowledge about available help resources in medical students compared to previous cohorts that did not participate in the seminar (Hypotheses 1a–d). Furthermore, we predicted that the onsite seminar will be more effective (i.e., elicit more positive outcomes) than the online seminar (Hypothesis 2).

## Methods

### Participants

This study was a quasi-experimental intervention study with data being collected with online surveys from 2020 until 2023. Invitations to participate in the study were sent to 3,975 students of the Medical University of Vienna via email. All students of the first year of the curriculum received one email one week prior to their participation in the curriculum module that contained the seminar ‘Coping with stress’ (T_1_) as well as one day afterwards (T_2_). Every year, the curriculum module is held in spring and has a duration of approximately four weeks. The seminar ‘Coping with stress’ was conducted online in 2020 and 2021 due to the COVID-19 pandemic and onsite in 2022 and 2023. Approximately 670 students have participated in the seminar per year. With two cohorts of students participating in the onsite version of the seminar and two cohorts participating in the online version, a total of 2,680 students were enrolled in the seminar. Furthermore, in order to collect data from a control group, we sent emails with invitations to participate in the study to all students who completed the curriculum module before 2018, i.e., the year the seminar ‘Coping with stress’ was implemented in the curriculum. These are cohorts who started their medical studies in the winter semester of 2015/2016 and 2016/2017 and were not enrolled in ‘Coping with stress’ or any other similar seminar. As a result, these students were already more advanced in their studies than those in the interventions groups when they participated in the current study. The invitation emails of the control group for T_1_ and T_2_ were sent on the exact same days as the emails to the intervention group in 2020 (i.e., the first cohort that participated in the seminar and was enrolled in the intervention group of the current study).

### Power analysis

We used G*Power version 3.1.9.7 [25] to conduct a sample size calculation. An ANOVA model with three experimental conditions (i.e., onsite intervention, online intervention, control group) and two measurements (i.e., T_1_, T_2_) with an assumed correlation of 0.70 (Schaufeli et al., 2019) will require a minimum of *n* = 135 participants in order to detect a medium-sized treatment effect of *f* = 0.25.

### Materials

Participants in all experimental conditions consisted of two specific cohorts of students of the Medical University of Vienna. Participants of Intervention group #1 (onsite intervention) were students who started their medical studies in the winter semester of 2021/2022 or 2022/2023 and participated in the seminar ‘Coping with stress’ onsite in May/June of 2022 or 2023. The seminar was centered around a presentation that covered stress and stress reactions, warning signals of experiencing too much stress, description of burnout and its prevalence among medical doctors and medical students, coping with stress and stress competence, as well as suicidal ideation and suicidal behavior among medical doctors. The presentation also provided a detailed overview of help resources for students, particularly of resources at the Medical University of Vienna.

The seminar also involved two group activities. First, at the beginning of the seminar, the students discussed in groups of two what they knew about burnout, which was then subsequently shared with the entire class and summarized and commented by the instructor. The second group activity was a key element of the seminar. The instructor asked the students to share difficulties students typically experience in the first year of the medical curriculum with the entire class. Afterwards, the instructor asked the students to share what had helped them overcoming these or other difficulties in the past and summarized and discussed the students’ coping strategies and solutions. Furthermore, in a practical exercise of personal self-reflection, the students were asked to write down (1) their strengths in terms of stress management, (2) areas of their stress competence they felt were already strong or needed improvement, (3) what they would like to learn in order to improve their stress competence, and (4) what they would personally like to take away from the seminar. At the conclusion of the seminar, the students received a brief questionnaire for self-assessment of risk of burnout and a brochure with an overview of available help resource for students, with a focus on those specifically offered for students of the Medical University of Vienna.

Participants of Intervention group #2 (online intervention) were students who started their medical studies in the winter semester of 2019/2020 or 2020/2021 and, due to the COVID-19 pandemic, participated in an online homeschooling version of the seminar ‘Coping with stress’ in April/May of 2020 or May/June of 2021. The seminar was organized via the learning management system *Moodle*. The students were instructed to write a short essay consisting of approximately 500 words on 1) what they knew or had heard about burnout and 2) what they considered to be typical difficulties in the first year of the medical curriculum and what students could do about them or which coping strategies were helpful. Each student received a short feedback on their essay by their instructor along with some additional potential coping strategies. The presentation slides used for the onsite seminars, a brief questionnaire for self-assessment of risk of burnout, and a brochure with an overview of available help resource for (medical) students were made available online.

Participants of the control group were students who started their medical studies in the winter semester of 2015/2016 or 2016/2017. At that time, the seminar ‘Coping with stress’ was not implemented in the curriculum, yet. Thus, these students completed the first year of the curriculum without participating in any version of this particular or any other similar seminar.

### Procedure

Data was collected with the intra-university online system *MedCampus*. After participants clicked on the link provided in their first invitation email at T_1_, they accessed the online survey, and informed consent was obtained in the first section of the survey. Afterwards, data on socio-demographics and all outcomes were collected. Emails with a link to a survey collecting all outcomes again were sent at T_2_. In the last section of both surveys, contact information of professional help organizations for individuals in crisis were provided. The participants had one week to complete each survey. Reminders were sent out by email three days and one day before each survey closed.

### Primary outcome measures

#### Burnout risk

We used the Burnout Assessment Tool (BAT) [26] to assess participants’ burnout risk, which was translated into German with the back-translation method (i.e., it was translated into German by the first author of the manuscript and then translated back into English by an independent translator who was blinded to the original questionnaire). The items were slightly adapted in order to fit in the university context (e.g., we replaced “at work” with “in class” or “at the university”). The BAT is a self-report measure that consists of four subscales: Exhaustion, measured with eight items (e.g., “At the end of my day, I feel mentally exhausted and drained”), mental distance, measured with four items (e.g., “I feel indifferent about my studies”), emotional impairment, measured with five items (e.g., “In class, I feel unable to control my emotions”), and cognitive impairment, measured with five items (e.g., “I’m forgetful and distracted in class”). All items are rated on a scale from 1 (*never*) to 5 (*always*). For each subscale, we calculated mean scores across the respective number of items, resulting in a score range of 1–5 (exhaustion: *M* = 2.14; *SD* = 0.64; α = 0.87; mental distance: *M* = 1.40; *SD* = 0.43; α = 0.57; emotional impairment: *M* = 1.34; *SD* = 0.44; α = 0.70; cognitive impairment: *M* = 1.77; *SD* = 0.67; α = 0.87). Higher scores on each subscale indicate greater burnout risk with regard to the respective aspect of burnout.

### Secondary outcome measures

#### Life satisfaction

The German version [27] of the Satisfaction with Life Scale [28] is a self-report measure consisting of five items that assess respondents’ life satisfaction (e.g., “I am satisfied with my life”). The respondents rate the items on a scale from 1 (*strongly disagree*) to 7 (*strongly agree*). Schumacher [27] reported satisfactory psychometric properties of the German version of this instrument in terms of item–total correlation, internal consistency reliability, and concurrent validity. We calculated mean scores across all five items of the scale (score range: 1–7; *M* = 5.38; *SD* = 1.19; α = 0.88), with higher scores indicating greater life satisfaction.

#### Stress

We used the subscale *Physical and psychological stress symptoms* of the Stress and Coping Inventory by Satow [29] to assess participants’ current level of stress. This self-report measure consists of 13 items (e.g., “I have trouble concentrating”), which are rated on a scale ranging from 1 (*does not apply at all*) to 4 (*fully applies*). The scale has satisfactory psychometric properties in terms of internal consistency reliability, split-half reliability, and concurrent validity [29]. We calculated mean scores across all 13 items of the scale (score range: 1–4; *M* = 1.99; *SD* = 0.60; α = 0.86), with higher scores indicating greater levels of stress.

#### Knowledge about help resources

As an indicator for participants’ knowledge about available help resources, we provided a list of 20 different help resources available for students of the Medical University (e.g., “Junior mentoring”, “Supervision for students in clinical practice”, or “Psychological Student Counseling Vienna”). We asked participants to indicate for each item if they were aware of the availability of the respective help resource (1 = *yes*, 0 = *no*). For each participant, the number of known help resources was then divided by the total number of items (i.e., 20). This resulted in a mean score (score range: 0–1; *M* = 0.44; *SD* = 0.17; α = 0.89), with higher scores indicating greater knowledge about available help resources.

### Data analysis

The four subscale scores of burnout risk as well as life-satisfaction, stress, and knowledge about help resources were each subjected to a repeated measures analysis of variance (ANOVA) with time (i.e., pre-exposure T_1_, post-exposure T_2_) used as within-subjects factor and experimental condition (i.e., onsite intervention, online intervention, control group) used as between-subjects factor. The Greenhouse-Geisser statistic was used to assess the statistical significance of the observed effects and interactions. Differences between individual groups (i.e., mean differences between T_2_ and T_1_ within each group as well as mean differences at T_2_ between individual experimental conditions) were examined with Bonferroni-corrected contrast tests. All analyses were controlled for gender and age. Gender and age are well-known factors that influence mental health in college and university students [16, 30–32]. Furthermore, with experimental conditions consisting of different cohorts of students in the current study, there were significant age differences between participants of the three experimental conditions, χ^2^ (8, *N* = 137) = 29.17, *p* <.001. Differences between study completers and dropouts were examined with chi-squared tests and two-sample t-tests.

## Results

In total, *n* = 324 individuals accessed the survey at T_1_, *n* = 379 individuals accessed the survey at T_2_, and *n* = 138 individuals accessed the survey at both points in time. With repeated measures ANOVAs being used for statistical analysis in the current study, only the *n* = 138 participants who accessed the survey at both, T_1_ and T_2_ were included in the data analysis. Due to the low number of observed frequencies, one individual who indicated “other gender” (*n* = 1) was excluded from statistical analysis. Thus, a total of *n* = 137 participants were included in the final statistical data analysis. This corresponds to 3.4% of students who were invited to participate in the study. Of these *n* = 137 participants, 36.5% (*n* = 50) completed the onsite seminar, 46% (*n* = 63) completed the online seminar, and 17.5% (*n* = 24) were in the control group. Thus, with approximately 2,680 students being enrolled in one of the two versions of the seminar since the start of this study in 2020, approximately 4.2% of these students were included in the data analysis. Furthermore, of the *n* = 137 participants, 32.1% were male (*n* = 44) and 67.9% were female (*n* = 93). In terms of age, 43.8% were 18–20 years (*n* = 60), 46.0% were 21–25 years (*n* = 63), 7.3% were 26–30 years (*n* = 10), 1.5% were 31–35 years (*n* = 2), and 1.5% were 41 years or older (*n* = 2).

### Comparisons of respondents who completed T_1_ and T_2_ of the survey with respondents who completed either T_1_ or T_2_

There were no differences between participants who have completed the survey at both T_1_ and T_2_ and those who only participated at T_1_ in terms of gender, χ^2^ (1, *N* = 320) = 0.29, *p* =.59, age group, χ^2^ (4, *N* = 320) = 3.67, *p* =.45, as well as T_1_ scores for exhaustion, *t*(312) = 1.94, *p* =.053, life satisfaction, *t*(314) = -0.31, *p* =.754, stress, *t*(313) = -0.32, *p* =.751, and knowledge about help resources, *t*(238) = 0.73, *p* =.464. However, burnout scores for mental distance, *t*(310) = 2.42, *p* =.016, emotional impairment, *t*(313) = 2.15, *p* =.033, and cognitive impairment, *t*(316) = 2.34, *p* =.020, were higher in students who dropped out after T_1_ than in study completers. Furthermore, there were no differences between participants who have completed the survey at both T_1_ and T_2_ and those who only participated at T_2_ in terms of T_2_ scores for exhaustion, *t*(314) = 1.08, *p* =.280, emotional impairment, *t*(374) = 0.71, *p* =.481, cognitive impairment, *t*(374) = 0.93, *p* =.353, life satisfaction, *t*(373) = -1.35, *p* =.179, and stress, *t*(368) = -1.06, *p* =.288. However, mental distance was higher, *t*(339) = 2.30, *p* =.022, and knowledge about help resources was lower, *t*(254) = -1.98, *p* =.049, at T_2_ in individuals who have only participated in the survey at T_2_ than in those who completed the survey at both T_1_ and T_2_.

### Effects on burnout risk

See Table 1 for an overview of means and corresponding 95% confidence intervals of all outcomes in each experimental condition before (T_1_) and after the intervention (T_2_) along with comparison of means between T_1_ and T_2_ within each experimental condition and of means at T_2_ between individual experimental conditions with Bonferroni corrected contrast tests. There was a small-sized significant interaction of time and experimental condition on emotional impairment, *F*(2, 131) = 3.32, *p* =.039, η_p_^2^ = 0.048. Results from Bonferroni corrected contrast tests indicated that emotional impairment decreased after the onsite intervention, *p* =.038, *d* = -0.18 (-0.35, -0.01), but did not change after the online intervention, *p* =.797, *d* = -0.02 (-0.19, 0.15), or in the control group, *p* =.098, *d* = 0.14 (-0.03, 0.31). There were also small interactions of time and experimental condition on exhaustion, *F*(2, 130) = 2.36, *p* =.098, η_p_^2^ = 0.035, and mental distance, *F*(2, 129) = 2.79, *p* =.065, η_p_^2^ = 0.042, close to statistical significance. Scores of exhaustion, *p* =.002, *d* = -0.27 (-0.44, -0.10), and mental distance, *p* =.021, *d* = -0.20 (-0.37, -0.03), decreased significantly after the onsite intervention, but did not change after the online intervention (exhaustion: *p* =.381, *d* = -0.07 [-0.24, 0.09]; mental distance: *p* =.426, *d* = 0.07 [-0.10, 0.24]) or in the control group (exhaustion: *p* = .928, *d* = 0.01 [-0.16, 0.18]; mental distance: *p* = .824, *d* = -0.02 [-0.15, 0.19]). There were no significant main effects or interactions with regard to cognitive impairment.

**Table 1:** All outcome variables before (T_1_) and after (T_2_) the intervention

Study variable	Group	T_1_ Mean score (95% CI)	T_2_ Mean score (95% CI)	Mean difference T_2_-T_1_	Mean difference at T_2_ compared to control group
*Primary outcomes*					
Burnout: Exhaustion (α = 0.87, *n*^a^ = 135)	Intervention group #1: Onsite intervention	2.27 (2.08–2.47)	2.07 (1.89–2.26)	**-0.21** **(-0.33– -0.08)**	0.18 (-0.24–0.59)
	Intervention group #2: Online intervention	2.12 (1.95–2.28)	2.07 (1.90–2.24)	-0.05 (-0.l6–0.06)	0.16 (-0.25–0.56)
	Control group	1.92 (1.72–2.13)	1.91 (1.67–2.14)	0.01 (-0.18–0.20)	–
Burnout: Mental distance (α = 0.57, *n*^a^ = 134)	Intervention group #1: Onsite intervention	1.52 (1.37–1.66)	1.40 (1.24–1.56)	**-0.12** **(-0.22– -0.02)**	-0.11 (-0.43–0.22)
	Intervention group #2: Online intervention	1.29 (1.22–1.36)	1.32 (1.21–1.43)	0.04 (-0.05 − 0.12)	-0.18 (-0.50–0.13)
	Control group	1.45 (1.23–1.67)	1.48 (1.24–1.72)	0.02 (-0.13–0.16)	–
Burnout: Emotional impairment (α = 0.70, *n*^a^ = 136)	Intervention group #1: Onsite intervention	1.38 (1.25–1.51)	1.30 (1.20–1.40)	**-0.09** **(-0.18– -0.01)**	-0.03 (-0.30–0.24)
	Intervention group #2: Online intervention	1.33 (1.21–1.45)	1.31 (1.20–1.43)	-0.01 (-0.09–0.07)	-0.02 (-0.28–0.25)
	Control group	1.26 (1.12–1.39)	1.36 (1.17–1.55)	0.11 (-0.02–0.24)	–
Burnout: Cognitive impairment (α = 0.87, *n*^a^ = 136)	Intervention group #1: Onsite intervention	1.92 (1.69–2.14)	1.81 (1.59–2.03)	-0.11 (-0.27–0.04)	0.15 (-0.32–0.62)
	Intervention group #2: Online intervention	1.72 (1.57 − 1.87)	1.80 (1.61–1.99)	0.09 (-0.05–0.23)	0.15 (-0.31–0.60)
	Control group	1.59 (1.37 − 1.82)	1.68 (1.42–1.95)	0.08 (-0.15–0.31)	–
*Secondary outcomes*					
Life satisfaction (α = 0.88, *n*^a^ = 136)	Intervention group #1: Onsite intervention	5.10 (4.73–5.46)	5.33 (4.96–5.70)	0.23 (-0.01–0.46)	-0.44 (-1.18–0.30)
	Intervention group #2: Online intervention	5.52 (5.23–5.81)	5.75 (5.48–6.03)	**0.23** **(0.02–0.44)**	-0.08 (-0.82–0.65)
	Control group	5.60 (5.18–6.02)	5.58 (4.99–6.16)	-0.02 (-0.38–0.33)	–
Stress (α = 0.86, *n*^a^ = 133)	Intervention group #1: Onsite intervention	2.09 (1.90–2.28)	1.89 (1.73–2.04)	**-0.21** **(-0.31– -0.10)**	0.20 (-0.15–0.55)
	Intervention group #2: Online intervention	1.95 (1.81–2.09)	1.95 (1.80–2.10)	0.00 (-0.09–0.10)	0.27 (-0.08–0.61)
	Control group	1.85 (1.59–2.11)	1.80 (1.54–2.05)	-0.06 (-0.22–0.10)	–
Knowledge about help resources (α = 0.74, *n*^a^ = 77)	Intervention group #1: Onsite intervention	0.44 (0.38–0.51)	0.58 (0.51–0.65)	**0.14** **(0.07–0.21)**	0.09 (-0.06–0.24)
	Intervention group #2: Online intervention	0.42 (0.36–0.48)	0.55 (0.48–0.62)	**0.13** **(0.07–0.19)**	0.06 (-0.09–0.21)
	Control group	0.51 (0.42–0.60)	0.51 (0.43–0.60)	-0.01 (-0.10–0.09)	–

Values are means with 95% confidence intervals given in parentheses, comparison of means between T_2_ and T_1_ within each experimental condition and of means at T_2_ between individual experimental conditions with Bonferroni corrected contrast tests as well as lower-bound (Cronbach α) sample reliabilities for each outcome. Mean differences with significant *p* values (< 0.05) are in bold

^a^Number of individuals included in the ANOVA

### Effects on secondary outcomes

There was a significant medium-sized interaction effect with regard to stress, *F*(2, 128) = 4.43, *p* =.014, η_p_^2^ = 0.065. Whereas stress decreased after the onsite intervention, *p* <.001, *d* = -0.33 (-0.50, -0.16), there was no significant change after the online intervention, *p* =.940, *d* = 0.01 (-0.16, 0.17), and in the control group, *p* =.452, *d* = -0.06 (-0.23, 0.10). Furthermore, there was a medium-sized significant interaction of time and experimental condition on knowledge about help resources, *F*(2, 72) = 3.74, *p* =.029, η_p_^2^ = 0.094. The contrast tests revealed that knowledge about help resources increased significantly after the onsite intervention, *p* <.001, *d* = 0.36 (0.19, 0.53), as well as the online intervention, *p* <.001, *d* = 0.38 (0.21, 0.55), but remained unchanged in the control group, *p* =.875, *d* = -0.01 (-0.18, 0.16). There were no significant main effects or interactions with regard to life satisfaction.

## Discussion

This study aimed to examine the effectiveness of a seminar in an obligatory undergraduate medical curriculum that was developed to reduce distress and risk of burnout in medical students and improve their mental health literacy and coping strategies in order to better prepare them for their professional career. First, we hypothesized that the seminar would reduce burnout risk, increase life satisfaction, decrease stress, and improve knowledge about available help resources in medical students. The results showed that, consistent with this hypothesis, medical students’ stress reduced and knowledge about help resources increased after participation in the onsite seminar ‘Coping with stress’, whereas no change was reported in the control group. These findings concur with results of several previous studies that found a reduction in stress and improvement in mental health after participation in stress management trainings [5, 19–23]. Importantly, the current seminar appears to be the first one that additionally improved some aspects of burnout risk in participants such as emotional impairment, exhaustion, and mental distance.

The positive effects of the current onsite seminar were small, and there were no significant differences in outcomes at T_2_ compared to the control group. In other words, mental health outcomes improved within the group of participants in the onsite seminar, but this change was not significantly different from the control group. It is, however, important to note that, with a singular session of 90 min, the duration was considerably shorter than the training evaluated in previous studies, which consisted of 4–10 weekly sessions ranging from 1.5 to 6 h [5, 18]. Thus, considering the short duration of the current intervention, the onsite seminar can be considered an important first step to help reduce stress and improve mental health in medical students. The present findings suggest that stress management training beyond primarily mindfulness-based trainings analyzed in previous studies [5, 18] can be a useful tool to reduce this risk in medical students during their training.

Furthermore, we predicted that the onsite seminar will elicit more positive outcomes than the online seminar. This hypothesis was supported by our findings. Whereas the online seminar increased participants’ knowledge about help resources, none of the other outcomes showed any improvement in that group. This finding differs from a previous study, which indicated that an online program for training in stress management and mental health literacy reduced university students’ anxiety and improved learning behavior [33]. Furthermore, there is some evidence for beneficial effects of web-based interventions for the management of stress in the workplace [34]. All of these programs, however, consisted of various modules and lasted several weeks [33, 34]. Based on the current findings, it appears that a brief online seminar might be useful for promoting available help services, but does not reduce stress or burnout risk in medical students.

The main differences of the onsite seminar as compared to the online seminar were the social interactions with other students and the group activities during the seminar. For example, in the onsite seminar, the students had the opportunity to discuss typical adversaries of medical students in the first year and how to overcome them and share experiences and knowledge about burnout. In the online seminar, the students were instructed to think about these topics all by themselves and write down their thoughts in an essay. Another difference was that the students received information materials about burnout and coping with stress in the online seminar, but they did not have an instructor explaining these information, discussing individual options, and answering any remaining questions. It seems that social interactions not only between the individual students, but also between students and instructor may be a key element for the reduction of stress and burnout risk in medical students. Future studies could investigate the impact of individual elements or components of these social interactions in order to identify how seminars on coping with stress in medical students need to be adapted to maximize beneficial effects.

It is interesting to note that stress and some aspects of burnout risk tended to be lower in the control group than in both intervention groups, but this difference was not statistically significant. A potential reason for these slight differences may be that the intervention groups consisted of students in their first year of their studies. As a result, they may have currently faced more challenges and were confronted with more new situations and tasks than students who were already more advanced in their studies (i.e., participants of the control group). Thus, we cannot rule out that the positive effects of the seminar found in the current study may be even more pronounced (as indicated by significant differences in outcomes at T_2_ between interventions groups and control group) if the participants of the control group might have been in a similar stage of their academic life as those in the intervention groups.

What can medical schools and other colleges, universities, and educational institutions learn from our findings? Implementing a seminar on improving mental health literacy and coping strategies to the first year of the obligatory part of the curriculum can have beneficial effects on students’ experienced distress and burnout risk. These seminars do not necessarily have to be long or repetitive and do not necessarily have to be primarily based on mindfulness training. In the current study, single lectures with a duration of 90 min that used predominantly conventional teaching techniques such as whole-class lectures and teacher-led instruction had a small beneficial effect on students’ mental health by reducing burnout risk and stress and increasing knowledge about available help resources. Social interactions with the lecturer and other students and group activities (e.g., discussion about typically experienced adversaries in the first year of the medical curriculum and how to overcome them) appeared to be key components for the beneficial impact of the seminar, as most positive effects could not be observed when students participated in an online version of the seminar that lacked these social interactions and group activities. It seems likely that the observed effects are note restricted to medical students, but also apply to other student populations. Future studies, however, are necessary to investigate this.

### Strengths and limitations

A strength of the current study was that we examined the impact of a seminar that was implemented in the first year of the undergraduate medical curriculum and was therefore obligatory for all students. Thus, participants in the seminar not only included students who already had the intention to improve their mental health literacy, but the entire cohort. Many medical curricula offer mental health trainings only on a voluntary basis and thus reach only a pre-selected group. Accordingly, previous studies have only tested the effectiveness of trainings that were voluntary or optional [5, 18–22].

There are, however, also several limitations. First, there was a high number of respondents who only participated either at T_1_ or T_2_ of the study and thus could not be included in the statistical data analysis. Moreover, these respondents partly reported less favorable mental health outcomes than those who completed the survey at both T_1_ and T_2_. This might indicate an attrition or self-selection bias, i.e., individuals who experienced less symptoms of burnout may have attributed this to their participation in the seminar and were thus more motivated to participate in the current study. Furthermore, due to the implementation of the seminar in the curriculum and the recruitment of entire cohorts of medical students, we were unable to randomize participants to the experimental conditions of the study. Another limitation is that, due to outcome measures being collected immediately before and after the intervention, we were unable to assess any positive effects of the seminar over longer time periods. Future studies assessing any mid- or long-term effects of ‘Coping with stress’ or similar seminars in the medical curriculum, for example by assessing the same outcomes at the end of the subsequent semesters or years, are warranted. The onset of the COVID-19 pandemic in 2020 and the implementation of lockdowns and other restrictions as well as changes in these restrictions between 2020 and 2023 may be another limitation of the current study. It may be the case that mental health of medical students varied between different phases of the pandemic (e.g., lockdown vs. no lockdown), which may have impacted the effects of the seminar. For example, a multi-wave cross-sectional survey from Austria has shown that mental health in the population deteriorated during the pandemic, particularly among young people, and that the perceived burden from COVID-19-related measures decreased continuously from spring to summer [35]. This trend or other temporary changes in COVID-19 restrictions could have influenced the positive effect found in the onsite seminar group. Furthermore, the reliability of the items measuring the burnout component of mental distance was relatively low (see Table 1), which is a psychometric limitation often found in scales with a small number of items in specific subscales [36]. Moreover, any other psychometric properties beyond the internal consistency reliability of this version of the Burnout Assessment Tool [26], which was translated and adapted specifically for the current study, were not assessed.

## Conclusion

In the current study, participants in the onsite seminar ‘Coping with stress’ showed improvements in terms of an increased knowledge about available help resources and reduced stress, and they showed partly decreased symptoms of burnout. We provided tentative evidence showing that the implementation of such a seminar in the medical curriculum might help to improve mental health in medical students. These findings are encouraging and suggest that integrating stress management trainings in medical curricula might be a useful approach in reducing burnout risk. Providing the same information in an online or distance learning setting, however, appears appeared to be less effective. Future studies comparing the effectiveness of different types of stress management interventions are warranted. It is, however, important to note that the implementation of interventions such as this seminar is only one component of stress reduction and burnout prevention in medical students [12]. Considering the high risk of burnout and suicidal ideation in medical students [9–12], structural adaptions in the medical curriculum, such as the implementation of systems to identify students at risk of suicide, student support, and wellness programs as well as the optimization of learning environments, clinical rotations, and the diversity of clinical experiences, are necessary in addition to the implementation of individual trainings and seminars in order to improve mental health in medical students [11].

## Data Availability

The data that support the findings of this study are available from the corresponding author upon reasonable request.

## References

[1] Krasner MS, Epstein RM, Beckman H, Suchman AL, Chapman B, Mooney CJ (2009). Association of an educational program in mindful communication with burnout, empathy, and attitudes among primary care physicians. JAMA.

[2] Rotenstein LS, Torre M, Ramos MA, Rosales RC, Guille C, Sen S (2018). Prevalence of burnout among physicians: a systematic review. JAMA.

[3] Maslach C, Jackson SE (1981). The measurement of experienced burnout. J Occupat Behav.

[4] Shanafelt TD, Boone S, Tan L, Dyrbye LN, Sotile W, Satele D (2012). Burnout and satisfaction with work-life balance among US physicians relative to the general US population. Arch Intern Med.

[5] de Vibe M, Solhaug I, Tyssen R, Friborg O, Rosenvinge JH, Sorlie T (2013). Mindfulness training for stress management: a randomised controlled study of medical and psychology students. BMC Med Edu.

[6] Schernhammer ES, Colditz GA (2004). Suicide rates among physicians: a quantitative and gender assessment (meta-analysis). AM J Psychiatry.

[7] Zimmermann C, Strohmaier S, Niederkrotenthaler T, Thau K, Schernhammer E (2023). Suicide mortality among physicians, dentists, veterinarians, and pharmacists as well as other high-skilled occupations in Austria from 1986 through 2020. Psychiatry Res.

[8] Firth-Cozens J (1998). Individual and organizational predictors of depression in general practitioners. Br J Gen Pract.

[9] Almutairi H, Aslubaiei A, Abduljawad S, Alshatti A, Fekih-Romdhane F, Husni M (2022). Prevalence of burnout in medical students: a systematic review and meta-analysis. Int J Soc Psychiatry.

[10] Steiner-Hofbauer V, Holzinger A (2020). How to cope with the challenges of medical education? Stress, depression, and coping in undergraduate medical students. Acad Psychiatry.

[11] Dyrbye LN, Thomas MR, Massie FS, Power DV, Eacker A, Harper W (2008). Burnout and suicidal ideation among U.S. medical students. Ann Intern Med.

[12] Dyrbye LN, West CP, Satele D, Boone S, Tan L, Sloan J (2014). Burnout among U.S. medical students, residents, and early career physicians relative to the general U.S. population. Acad Med.

[13] Dyrbye LN, Shanafelt T (2016). A narrative review on burnout experienced by medical students and residents. Med Educ.

[14] Mazurkiewicz R, Korenstein D, Fallar R, Ripp J. The prevalence and correlations of medical student burnout in the pre-clinical years: a cross-sectional study. Psychol Health Med 201217(2):188–95. 10.1080/13548506.2011.597770 21781020

[15] Dunn LB, Iglewicz A, Moutier C. (2008). A conceptual model of medical student well-being: Promoting resilience and preventing burnout. Acad Psychiatry. 2008;32(1):44–53. 10.1176/appi.ap.32.1.44 18270280

[16] Kjeldstadli K, Tyssen R, Finset A, Hem E, Gude T, Gronvold NT (2006). Life satisfaction and resilience in medical school– a six-year longitudinal, nationwide and comparative study. BMC Med Educ.

[17] Oliva Costa EF, Santos SA, Abreu Santos AT, Melo EV, Andrade TM. Burnout syndrome and associated factors among medical students: a cross-sectional study. Clinics. 201267(6):573–9. 10.6061/clinics/2012(06)05 PMC3370307 22760894

[18] Kuhlmann SM, Huss M, Bürger A, Hammerle F (2016). Coping with stress in medical students: results of a randomized controlled trial using a mindfulness-based stress prevention training (MediMind) in Germany. BMC Med Educ.

[19] Jain S, Shapiro SL, Swanick S, Roesch SC, Mills PJ, Bell I (2007). A randomized controlled trial of mindfulness meditation versus relaxation training: effects on distress, positive states of mind, rumination, and distraction. Ann Behav Med.

[20] Rosenzweig S, Reibel DK, Greeson JM, Brainard GC, Hojat M (2003). Mindfulness-based stress reduction lowers psychological distress in medical students. Teach Learn Med.

[21] Shapiro SL, Schwartz GE, Bonner G (1998). Effects of mindfulness-based stress reduction on medical and premedical students. J Behav Med.

[22] Shapiro SL, Brown KW, Biegel GM (2007). Teaching self-care to caregivers: effects of mindfulness-based stress reduction on the mental health of therapists in training. Train Educ Prof Psychol.

[23] Yusoff M (2014). Interventions on medical students’ psychological health: a meta-analysis. J Taibah Univ Med Sci.

[24] Tang R, Broderick PC, Bono T, Dvoráková K, Braver TS (2022). A college first-year mindfulness seminar to enhance psychological well-being and cognitive function. J Stud Aff Res Pract.

[25] Faul F, Erdfelder E, Lang AG, Buchner A (2007). G*Power 3: a flexible statistical power analysis program for the social, behavioral, and biomedical sciences. Behav Res Methods.

[26] Schaufeli W, De Witte H, Desart S (2019). Manual Burnout Assessment Tool (BAT).

[27] Schumacher J, Schumacher J, Klaiberg A, Brähler E (2003). SWLS – satisfaction with Life Scale. Diagnostische Verfahren zu Lebensqualität Und Wohlbefinden.

[28] Diener E, Emmons RA, Larsen RJ, Griffin S (1985). The satisfaction with Life Scale. J Pers Assess.

[29] Satow L. Stress- und coping-inventar (SCI) [Stress and coping inventory (SCI)]. Leibniz-Zentrum für Psychologische Information Und Dokumentation (ZPID), editor. Elektronisches Testarchiv. ZPID; 2012. PSYNDEX Test-Nr. 9006508.

[30] Campbell F, Blank L, Cantrell A, Baxter S, Blackmore C, Dixon J (2022). Factors that influence mental health of university and college students in the UK: a systematic review. BMC Public Health.

[31] Duffy A, Keown-Stoneman C, Goodday S, Horrocks J, Lowe M, King N (2020). Predictors of mental health and academic outcomes in first-year university students: identifying prevention and early-intervention targets. BJPsych Open.

[32] Zivin K, Eisenberg D, Gollust SE, Golbrstein E (2009). Persistence of mental health problems and needs in a college student population. J Affect Disord.

[33] Charbonnier E, Trémolière B, Baussard L, Goncalves A, Lespiau F, Philippe AG, Le Vigouroux S (2022). Effects of an online self-help intervention on university students’ mental health during COVID-19: a non-randomized controlled pilot study. Comput Hum Behav Rep.

[34] Ryan C, Bergin M, Chalder T, Wells JS (2017). Web-based interventions for the management of stress in the workplace: Focus, form, and efficacy. J Occup Health.

[35] Niederkrotenthaler T, Laido Z, Kirchner S, Braun M, Metzler H, Waldhör T (2022). Mental health over nine months during the SARS-CoV2 pandemic: Representative cross-sectional survey in twelve waves between April and December 2020 in Austria. J Affect Disord.

[36] Sijtsma K (2009). On the use, the misuse, and the very limited usefulness of Cronbach’s alpha. Psychometrika.

